# Receptor-Interacting Protein Kinase 3 Deficiency Delays Cutaneous Wound Healing

**DOI:** 10.1371/journal.pone.0140514

**Published:** 2015-10-09

**Authors:** Andrew Godwin, Archna Sharma, Weng-Lang Yang, Zhimin Wang, Jeffrey Nicastro, Gene F. Coppa, Ping Wang

**Affiliations:** 1 Department of Surgery, Hofstra North Shore-LIJ School of Medicine, Manhasset, New York, United States of America; 2 Center for Translational Research, The Feinstein Institute for Medical Research, Manhasset, New York, United States of America; Albert Einstein College of Medicine, UNITED STATES

## Abstract

Wound healing consists of a complex, dynamic and overlapping process involving inflammation, proliferation and tissue remodeling. A better understanding of wound healing process at the molecular level is needed for the development of novel therapeutic strategies. Receptor-interacting protein kinase 3 (RIPK3) controls programmed necrosis in response to TNF-α during inflammation and has been shown to be highly induced during cutaneous wound repair. However, its role in wound healing remains to be demonstrated. To study this, we created dorsal cutaneous wounds on male wild-type (WT) and RIPK3-deficient (*Ripk3* ^-/-^) mice. Wound area was measured daily until day 14 post-wound and skin tissues were collected from wound sites at various days for analysis. The wound healing rate in *Ripk3* ^-/-^ mice was slower than the WT mice over the 14-day course; especially, at day 7, the wound size in *Ripk3* ^-/-^ mice was 53% larger than that of WT mice. H&E and Masson-Trichrome staining analysis showed impaired quality of wound closure in *Ripk3* ^-/-^ wounds with delayed re-epithelialization and angiogenesis and defected granulation tissue formation and collagen deposition compared to WT. The neutrophil infiltration pattern was altered in *Ripk3* ^-/-^ wounds with less neutrophils at day 1 and more neutrophils at day 3. This altered pattern was also reflected in the differential expression of IL-6, KC, IL-1β and TNF-α between WT and *Ripk3* ^-/-^ wounds. MMP-9 protein expression was decreased with increased Timp-1 mRNA in the *Ripk3* ^-/-^ wounds compared to WT. The microvascular density along with the intensity and timing of induction of proangiogenic growth factors VEGF and TGF-β1 were also decreased or delayed in the *Ripk3* ^-/-^ wounds. Furthermore, mouse embryonic fibroblasts (MEFs) from *Ripk3* ^-/-^ mice migrated less towards chemoattractants TGF-β1 and PDGF than MEFs from WT mice. These results clearly demonstrate that RIPK3 is an essential molecule to maintain the temporal manner of the normal progression of wound closure.

## Introduction

Cutaneous wound healing is a complex and dynamic process involving multiple overlapping events following injury, including coagulation, inflammation, epithelialization, formation of granulation tissue, matrix deposition, and tissue remodeling [1–3]. Normal wound healing is orchestrated by an intricate system of growth factors, chemokines, cytokines, and angiogenic factors which bind to their specific receptors and activate and coordinate a complex network of various signal transduction pathways [4, 5]. The disruption of this tightly regulated cascade of events may lead to impaired or delayed healing of acute cutaneous wounds and to the development of chronic non-healing wounds/ulcers [6, 7]. Certain chronic conditions predispose patients to poor wound healing and these include diabetes mellitus, venous and arterial insufficiency and long standing immobility or hospitalization and subsequent development of pressure ulcers [8]. These chronic and slowly healing acute wounds account for significant levels of morbidity as well as severe decrease in quality of life and incur huge health care costs [9, 10]. A better understanding of the molecular mechanisms involved in wound healing is needed for the development of novel and effective therapies for patients with aberrant wound healing.

Receptor-interacting protein kinases (RIPKs) are a family of serine/threonine protein kinases containing seven members RIPK1-RIPK7, all with a relatively conserved N-terminal kinase domain but distinct non-kinase regions [11]. They associate with the intracellular domain of members of the tumor necrosis factor receptor (TNFR) family of proteins and mediate downstream signaling for the regulation of inflammation, immune responses and various death-inducing processes [12]. Particularly, RIPK4 has been shown as an important regulator of cutaneous wound repair as it regulates epidermal development and homeostasis [13–15]. Impaired and delayed wound healing has also been associated with increased TNF-α levels [16, 17]. Recently, RIPK3 has been shown to be strongly induced as early as 1 day after cutaneous wounding [13]. The C-terminal domain of RIPK3 functions as a molecular switch between TNF-α-induced apoptosis and programmed necrosis (necroptosis) [18–25]. It also participates in inflammasome activation and IL-1β cytokine production [26]. However, the precise role of RIPK3 in the wound healing process has not been elucidated yet.

In the present study, we used RIPK3-deficient (*Ripk3* ^-/-^) mice in a model of cutaneous wound healing to examine the role of RIPK3 in wound healing process. We first compared the wound closure in WT and *Ripk3* ^-/-^ mice over the 14-day course to determine whether RIPK3 deficiency altered the progression of wound healing. We then looked at the differences in the quality of wound closure between WT and *Ripk3* ^-/-^ mice by H&E and in collagen deposition by Masson-Trichrome staining. Next, we examined if RIPK3 deficiency affected the neutrophil trafficking as well as expression of pro-inflammatory cytokines during wound healing. We also assessed the expression of matrix metalloproteinase (MMP)-9, vascular endothelium growth factor (VEGF) and transforming growth factor (TGF) β-1 which are involved in inflammation, epithelialization, angiogenesis and matrix remodeling during different time frames in the wound healing process. Finally, we conducted an *in vitro* chemotaxis assay with embryonic fibroblasts (MEFs) isolated from WT and *Ripk3* ^-/-^ mice to determine the direct effect of RIPK3 deficiency on fibroblast migration. Our study showed that RIPK3 is required for normal progression of wound closure and its deficiency delays wound healing.

## Materials and Methods

### Mice

C57BL/6 mice were bred in our own facility including wild type (WT) control and *Ripk3* ^-/-^ mice which were obtained from V. Dixit (Genentech, San Francisco, CA, USA) [27]. Male WT and *Ripk3* ^-/-^ mice were used at the age of 10-12-weeks and housed in a temperature-controlled room on a 12 h light/dark cycle and fed a standard laboratory diet within the Feinstein Institute for Medical Research (Manhasset, NY, USA). All experiments were performed in accordance with the recommendations in the Guide for the Care and Use of Laboratory Animals of the National Institutes of Health and the protocol was approved by the Institutional Animal Care and Use Committee (IACUC) at the Feinstein Institute for Medical Research. All surgery was performed under isoflurane anesthesia, and all efforts were made to minimize suffering.

### Cutaneous wound model

Mice were anesthetized with isoflurane inhalation. Full-thickness 20 mm diameter circular excisional wounds were surgically created on the dorsal skin of WT and *Ripk3* ^-/-^ mice as previously described [28]. Briefly, the dorsum extending from the cervical to lumbar regions was shaved, followed by a 10% povidone-iodine wash and a local injection of 0.5% Lidocaine intradermally to the affected area prior to creation of the wound. The wounds were created and extended to the muscular layer excluding the *panniculus carnosus* by using scissors. Bleeding was stopped by compression with cotton sticks. Wounds were immediately covered with hydro-fiber dressing held in place by an adhesive bandage (Tegaderm) which was changed daily. Wound closure areas were photographed and measured daily during dressing changes until day 14 post-wound. At days 1, 3, 7, and 14 after wound injury batches of animals were euthanized by CO_2_ asphyxiation and full thickness skin samples were collected from the entire wound sites (including the scab and epithelial margins) for evaluation. Full thickness skin samples from sham-operated (non-wounded) mice served as pre-wound controls. The skin samples were divided in two halves- one half fixed and stored in 10% formalin for histological analysis and other half frozen in liquid nitrogen and stored at -80°C for qPCR analysis.

### Measurement of wound closure rate

The residual wound area was traced on a transparent film daily after skin excision until day 14 and the pixel of the traced area was analyzed by ImageJ (Software 1.48q, Rayne Rasband, National Institutes of Health, USA). Wound area analysis was done by a different person who was blinded to the groups. Wound area percentage was calculated according to the following formula: Wound area (%) = [(Area_day n_) /Area_day 0_] ×100; where Area_day 0_ is the initial wound area at day 0 and the Area_day n_ is the area on day n after wounding.

### Histological and immunohistochemistry evaluation

The wounded skin tissues were harvested and fixed in 10% formalin and later embedded in paraffin. The tissue blocks were then cut into 5 μm sections, transferred to glass slides, and stained with H&E or Masson-Trichrome. Morphologic alterations in the skin tissues were examined by light microscopy and documented by photographs. For immunostaining, paraffin-embedded sections were deparaffinized in xylene and rehydrated in a graded series of ethanol. Antigen retrieval was performed in the citrate-based antigen unmasking solution, pH 6 (Vector Laboratories, Burlingame, CA) at 95°C for 15 min. Endogenous peroxidase activity was quenched by exposing to 2% hydrogen peroxide in 60% methanol for 20 min. After blocking with 2% normal goat serum in Tris-buffered saline, the sections were incubated overnight with anti-mouse Gr-1 antibody (BioLegend, San Diego, CA) or anti-mouse MMP-9 antibody (Calbiochem, Gibbstown, NJ), or anti-mouse CD31 antibody (Santa Cruz Biotechnology, Dallas, TX), followed by biotinylated species-specific secondary antibody (Vector Laboratories, Burlingame, CA). The detection was carried out with VECTASTAIN Elite ABC reagent and DAB (3, 3’-diaminobenzidine) HRP (horseradish peroxidase) substrate kit (Vector Laboratories, Burlingame, CA) as per the manufacturer’s instructions and counterstained with hematoxylin. The immunostaining was examined under a Nikon Eclipse E600 microscope by at least two investigators blinded to the genotype. The number of Gr-1 positive neutrophils and CD31 positive blood vessels with a visible lumen were manually counted in a microscopic field centered on the wound site in the immunostained sections. Neutrophil infiltration and microvascular density were evaluated based on the average number of neutrophils or blood vessels per counting field from 3 wounds per group for each time point.

### qPCR analysis

Total RNA was extracted from skin tissues using TRIzol reagent (Invitrogen, Carlsbad, CA, USA) and was reverse-transcribed into cDNA using murine leukemia virus reverse transcriptase (Applied Biosystems, Foster City, CA, USA). A PCR reaction was carried out in a 24 μl final volume containing 0.08 μM of each forward and reverse primer, cDNA, and 12 μl SYBR Green PCR Master Mix (Life Technologies, Grand Island, NY). Amplification was conducted in an Applied Biosystems 7300 real-time PCR machine (Applied Biosystems) under the thermal profile of 50°C for 2 min and 95°C for 10 min, followed by 40 cycles of 95°C for 15 s and 60°C for 1 min. The data was analyzed by the 2^-ΔΔCt^ method for relative quantization normalized to mouse β-actin mRNA. Relative expression of mRNA was expressed as the fold change in comparison with the pre-wound or normal skin tissues. The primers used for this study are listed in Table 1.

**Table 1:** Sequences of the primers used in qPCR.

Gene name	Forward primer sequence	Reverse primer sequence
*Col1a1*	GCAAGAGGCGAGAGAGGTTT	GACCACGGGCACCATCTTTA
*Il6*	CCGGAGAGGAGACTTCACAG	GGAAATTGGGGTAGGAAGGA
*Cxcl1*	GCTGGGATTCACCTCAAGAA	ACAGGTGCCATCAGAGCAGT
*I11b*	CAGGATGAGGACATGAGCACC	CTCTGCAGACTCAAACTCCAC
*Tnfa*	AGACCCTCACACTCAGATCATCTTC	TTG CTACGACGTGGGCTACA
*Mmp9*	CATTCGCGTGGATAAGGAGT	ACTGCACGGTTGAAGCAAA
*Timp1*	GCAAAGAGCTTTCTCAAAGACC	AGGGATAGATAAACAGGGAAACACT
*Vegfa*	TTACTGCTGTACCTCCACC	ACAGGACGGCTTGAAGATG
*Tgfb1*	AGCCCGAAGCGGACTACTAT	CTCTGCACGGGACAGCAAT
*Actb*	CGTGAAAAGATGACCCAGATCA	TGGTACGACCAGAGGCATACAG

### Mouse embryonic fibroblasts isolation

Mouse embryonic fibroblasts (MEFs) were isolated from freshly dissected 13–14 days old embryos of WT and *Ripk3* ^-/-^ mice using aseptic techniques as per standard protocols. Briefly, head and red organs were removed from embryos and the remaining embryo-portions were finely minced with a sterile razor blade, followed by Trypsin-EDTA digestion at 37°C for 10–15 min. with repeated pipetting every 5 min. for cell dissociation. After trypsin inactivation, the single cell suspensions of MEFs were cultured in Dulbecco's modified Eagle's medium (DMEM) supplemented with 10% heat-inactivated FBS, 2 mM glutamine and antibiotics (100 U/ml penicillin, 50 μg/ml streptomycin) and split every 3–4 days.

### Chemotaxis assay

WT and *Ripk3* ^-/-^ MEFs (5 ×10^4^ cells/500 μl/well) in FBS-free DMEM were added to the cell culture inserts with 8.0 μm pore size (Corning, Durham, NC) placed in a 24 well plate. The lower chambers of 24-well plate contained FBS-free DMEM (700 μl/well) with 10 ng/ml of chemoattaractants- transforming growth factor (TGF)-β1 or platelet-derived growth factor (PDGF). The plate was incubated at 37°C in a 5% CO_2_ atmosphere for 16 h and then the upper surface of the filter was swabbed with cotton to remove non-migratory cells. Migrated cells were fixed with 10% formalin and stained with 1 μg/ml propidium iodide (PI). Four random microscopic fields per well were counted.

### Statistical analysis

Data are expressed as mean ± standard error mean (SEM) and analyzed using SigmaPlot11 graphing and statistical analysis software (Systat Software Inc., San Jose, CA, USA). Multiple groups were compared by one-way analysis of variance (ANOVA) using Student-Newman-Keuls’ (SNK) test. Student’s *t* test was used for two-group analysis. Differences in values were considered significant if *P* < 0.05.

## Results

### RIPK3 deficiency delays cutaneous wound healing

**Fig 1:**
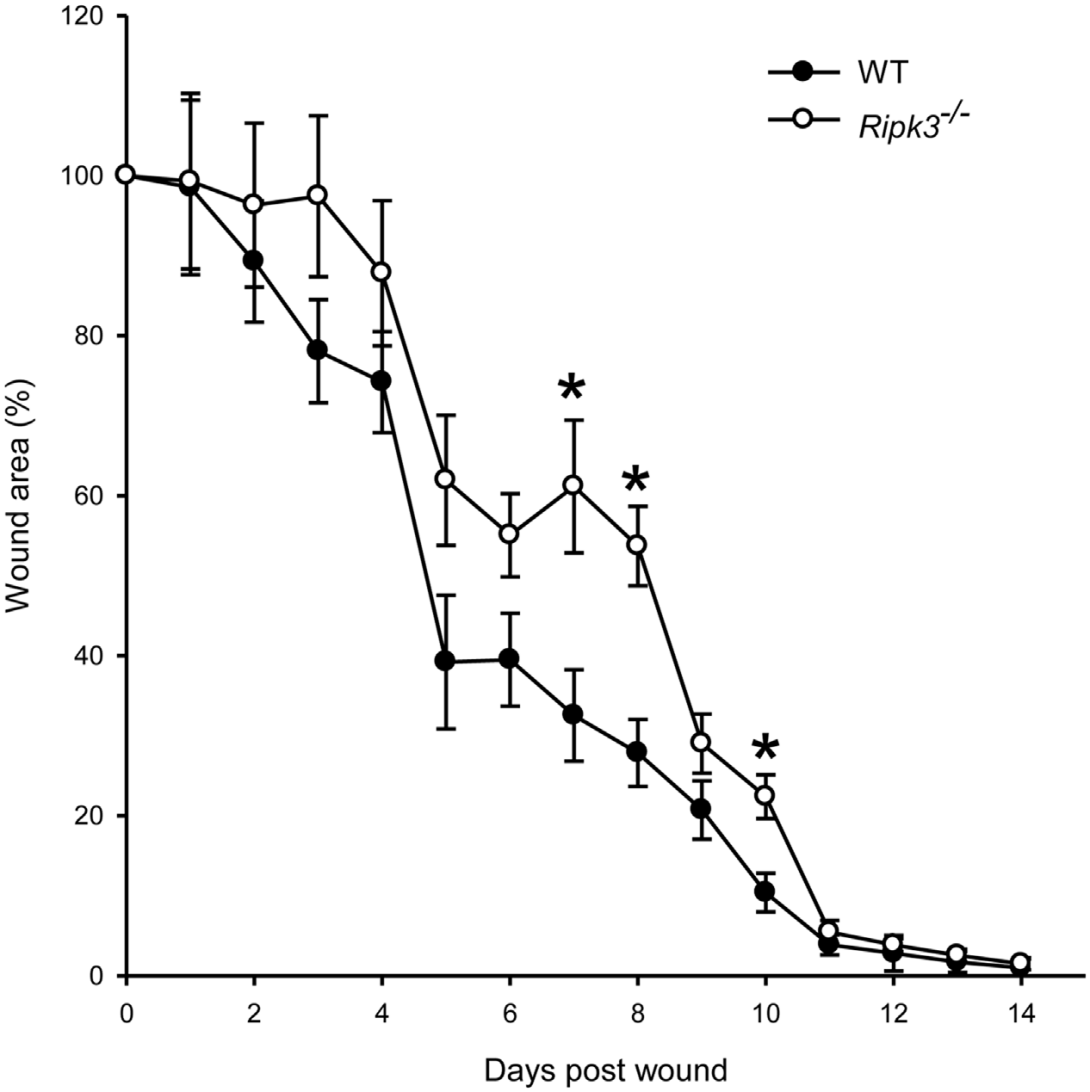
*Ripk3* ^-/-^ mice show delayed wound closure. WT (n = 7) and *Ripk3* ^-/-^ mice (n = 7) were subjected to dorsal cutaneous wounds as described in Materials and Methods for a 14-day wound healing study. Wound area percentage was determined based on measuring wound area daily using NIHImage J analysis as described in Materials and Methods. Data are represented as mean ± SEM and compared by one-way ANOVA using SNK test. *P < 0.05 versus WT at the indicated day.

We first compared a 14-day wound closure between WT and *Ripk3* ^-/-^ mice to determine whether RIPK3 deficiency altered the progression of wound healing. At day 7 post-wound, the wound area in *Ripk3* ^-/-^ group was 53% larger than that in WT group and remained significantly larger by 51% and 46% at days 8 and 10, respectively (Fig 1). This result indicates that the wound healing in *Ripk3* ^-/-^ mice group was slower compared to the WT mice group.

### RIPK3 deficiency impairs quality of wound closure

**Fig 2:**
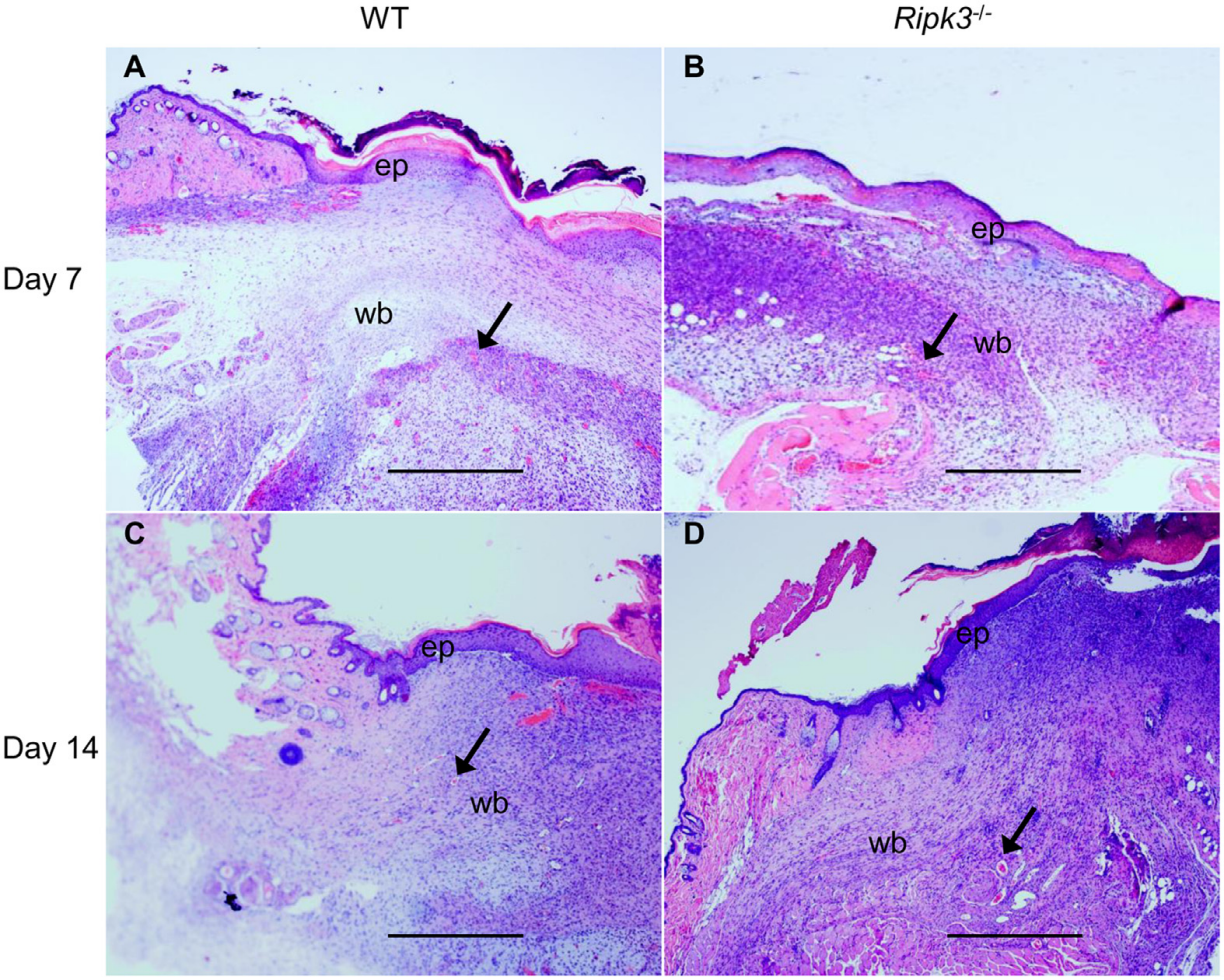
*Ripk3* ^-/-^ mice show impaired quality of wound closure. Skin samples from the wound sites of WT and *Ripk3* ^-/-^ mice were collected at days 7 and 14 for histological evaluation by H&E staining (n = 5 mice per group). Representative images of H&E stained skin sections (40×magnification) from day 7 (A, B) and day 14 (C, D) wounds from WT (A, C) and *Ripk3* ^-/-^ (B, D) mice are shown. Angiogenic sites are depicted by arrows; ep, new epithelium; wb, wound bed. Scale bar = 500 μm.

We then examined the effect of RIPK3 deficiency on the progression of wound healing to different phases at days 7 and 14 post-wound by H&E staining analysis. Angiogenesis was evident and re-epithelialization had begun in WT wounds by day 7 (Fig 2A) whereas it was not evident in *Ripk3* ^-/-^ wounds (Fig 2B). By day 14, WT wounds were well healed with re-epithelialization and decrease in overall cellularity and vascularization, characteristic of wound maturation (Fig 2C). WT wounds at day 14 also showed thick granulation tissue base with regular collagen deposition (Fig 2C). However, the granulation tissue in day 14 *Ripk3* ^-/-^ wounds was thin and irregular in shape compared to WT wounds, as well as displayed vascularization like WT wounds at day 7 (Fig 2D).

**Fig 3:**
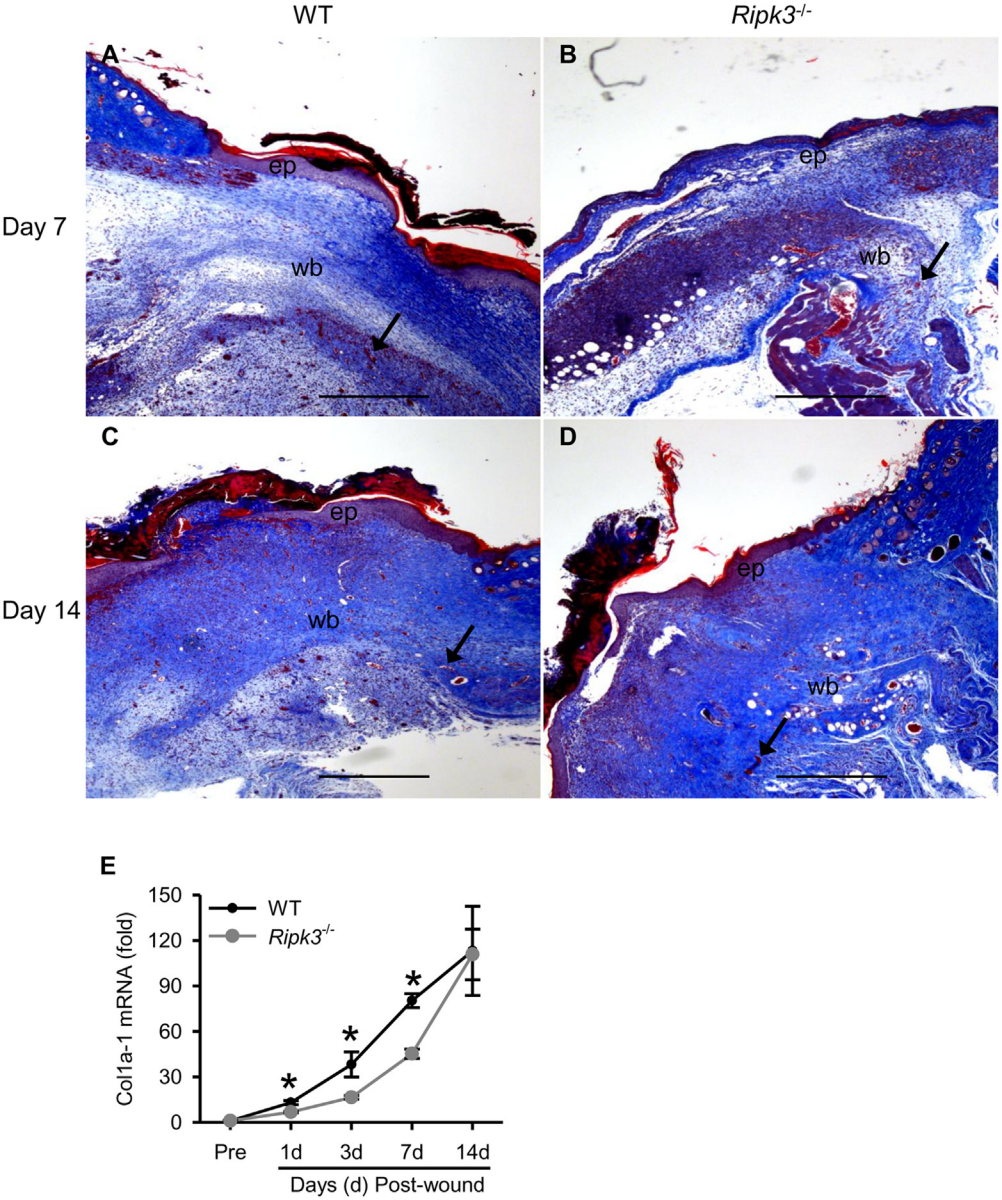
*Ripk3* ^-/-^ mice show impaired collagen deposition. Skin samples from the wound sites of WT and *Ripk3* ^-/-^ mice were collected at the indicated days for evaluation of collagen deposition by Masson-Trichrome staining and qPCR (n = 5 mice per group). Representative images of Masson-Trichrome stained skin sections (40× magnification) from day 7 (A, B) and day 14 (C, D) wounds from WT (A, C) and *Ripk3* ^-/-^ (B, D) mice are shown. Collagen is stained blue. Angiogenic sites are depicted by arrows; ep, new epithelium; wb, wound bed. Scale bar = 500 μm. (E) The expression analysis of collagen type 1 α 1 (*Col1a1*) mRNA by qPCR. Data expressed as mean ± SEM (n = 5 mice per group) and compared by one-way ANOVA using SNK test. *P < 0.05 versus WT at the indicated day.

By performing Masson trichrome staining which stains collagen blue, we observed that the overall collagen content in the *Ripk3* ^-/-^ wounds was lower than that in the WT wounds at day 7 (Fig 3A and 3B). However, the collagen content in day 14 *Ripk3* ^-/-^ wounds was similar to but less organized than WT wounds (Fig 3C and 3D). We also determined the gene expression of collagen type 1 (*Col1a-1*) which is highly induced at the late-phase of wound healing process. As shown in Fig 3E, mRNA levels of Colla-1 in *Ripk3* ^-/-^ wounds were 56.8% and 43.6% lower than those in WT wounds at day 3 and 7, respectively, while there was no difference between *Ripk3* ^-/-^ and WT wounds at day 14. Together, the data showed a qualitative impairment of the wound healing process in the *Ripk3* ^-/-^ mice, evidenced by delayed re-epithelialization and angiogenesis and defected granulation tissue formation and collagen deposition.

### RIPK3 deficiency alters early phase of wound associated inflammation

**Fig 4:**
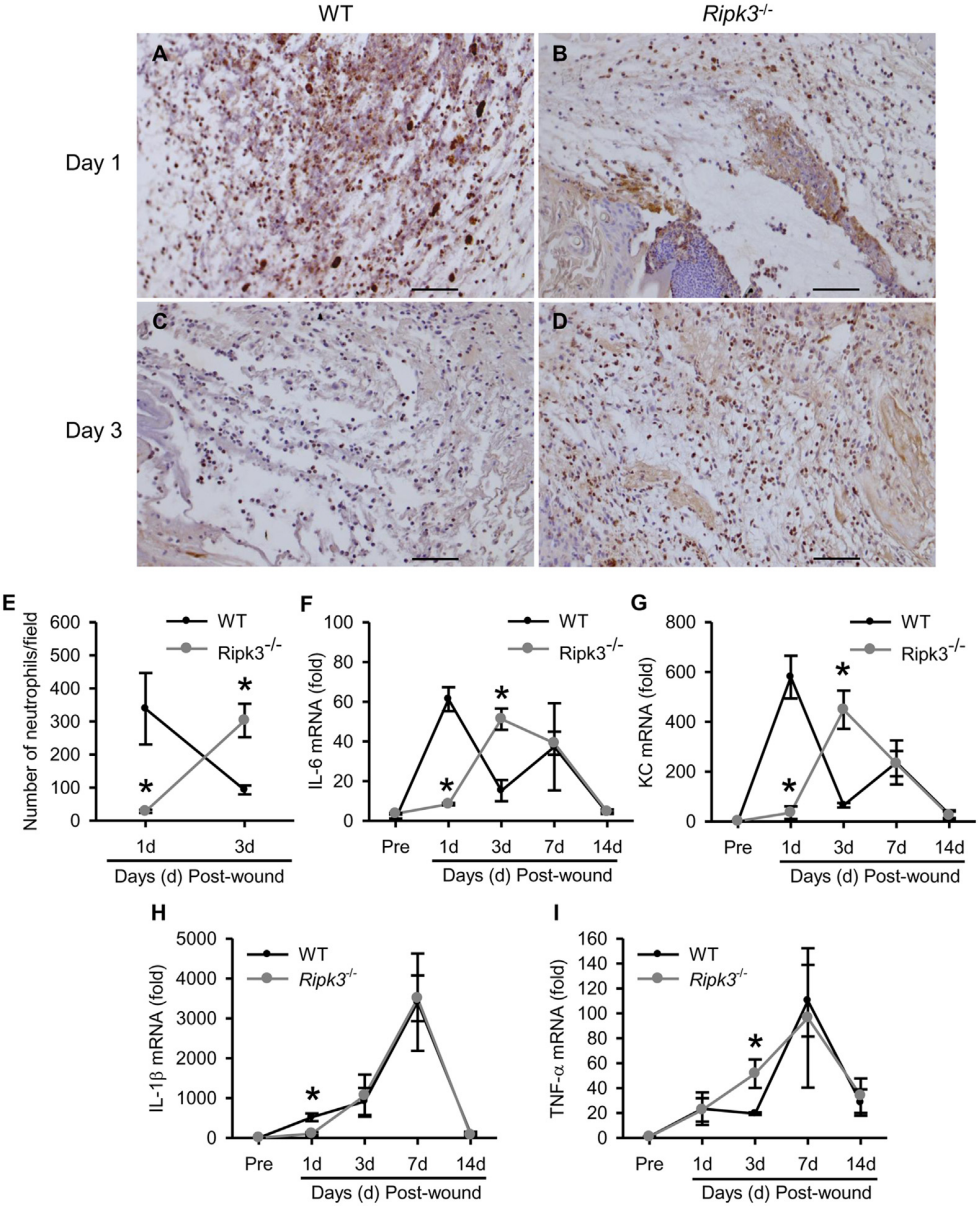
*Ripk3* ^-/-^ mice show altered early neutrophil infiltration and inflammation. (A-E) Skin samples from the wound sites of WT and *Ripk3* ^-/-^ mice were collected at days 1 and 3 for immunohistochemical staining with Gr-1 antibody. Representative images of Gr-1 stained skin sections (200× magnification) from day 1 (A, B) and day 3 (C, D) wounds from WT (A, C) and *Ripk3* ^-/-^ (B, D) mice and quantitative analysis of neutrophil infiltration expressed as number of neutrophils/field (E) are shown. Neutrophils are stained brown. Scale bar = 100 μm. (F-I) Skin samples from the wound sites of WT and *Ripk3* ^-/-^ mice were collected at the indicated days. RNA was extracted and cDNA was prepared from these samples for gene expression analysis of IL-6 (F), KC (G), IL-1β (H) and TNF-α (I) by qPCR. Data expressed as mean ± SEM (n = 3 (E) and 5 (F-I) mice per group for each time point) and compared by one-way ANOVA using SNK test. *P < 0.05 versus WT at the indicated day.

The inflammatory phase of the wound healing is marked by an infiltration of numerous leukocytes mainly neutrophils and induction of inflammatory cytokines. Neutrophil infiltration to the wounded skin typically occurs very early on and is essential for optimal wound healing. We assessed neutrophil infiltration by immunostaining the wounded skin tissues with neutrophil marker Gr-1. At day 1 post-wound, *Ripk3* ^-/-^ wounds demonstrated marked delay in neutrophil infiltration into the wounded tissue, with neutrophil numbers decreased by 91.7%, compared to wounds in WT control (Fig 4A, 4B and 4E). However, at day 3 post-wound, we observed a significant increase in neutrophil infiltration in *Ripk3* ^-/-^ wounds than that in WT wounds, with neutrophil numbers increased by 3.3-fold (Fig 4C, 4D and 4E). We further determined the expression levels of IL-6 and keratinocyte-derived chemokine (KC), which are two important factors in regulating neutrophil infiltration, by qPCR. The mRNA expression of IL-6 and KC in WT wounds showed biphasic induction peaking at days 1 and 7 (Fig 4F and 4G). At day 1, the mRNA levels of IL-6 and KC in WT wounds were 7.3- and 16.6-fold higher than those in *Ripk3* ^-/-^ wounds, respectively (Fig 4F and 4G). In contrast, mRNA levels of IL-6 and KC in *Ripk3* ^-/-^ wounds only showed a peak induction at day 3 with 3.4- and 6.9-fold higher levels than those in WT wounds, respectively (Fig 4F and 4G).

We further analyzed the inflammation status of the wounded skin by measuring gene expression levels of proinflammatory cytokines IL-1β and TNF-α, using qPCR. At day 1 the IL-1β mRNA levels was increased by 500-fold in WT wound tissue compared to pre-wound levels. In contrast, at day 1 there was only 100-fold increase in IL-1β levels in *Ripk3* ^-/-^ wound tissue compared to pre-wound levels which comes to a significant 80% decrease compared to WT wounds (Fig 4H). In addition, at day 3 the TNF-α levels in the *Ripk3* ^-/-^ wounds were significantly higher by 2.6-fold compared to WT wounds (Fig 4I). By day 7 till day 14, IL-6, KC, IL-1β and TNF-α levels were comparable between WT and *Ripk3* ^-/-^ wounds, high at day 7 and then returning close to pre-wound levels by day 14 (Fig 4F–4I). These data suggest that RIPK3 deficiency altered the timing of neutrophil infiltration and inflammatory cytokine production during the wound healing.

### RIPK3 deficiency decreases MMP-9 expression

**Fig 5:**
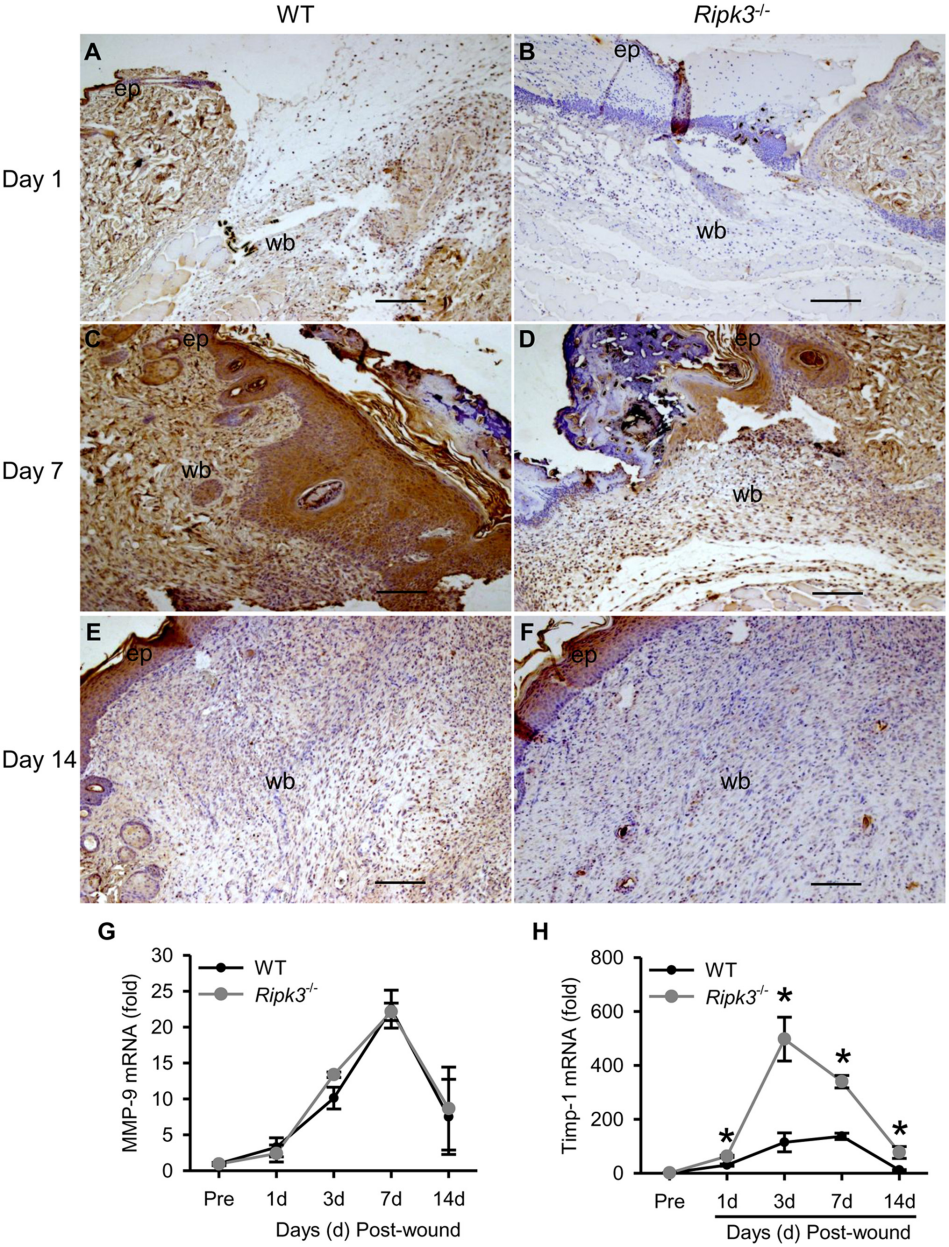
*Ripk3* ^-/-^ mice show reduced MMP-9 levels. Skin samples from the wound sites of WT and *Ripk3* ^-/-^ mice were collected at days 1, 7 and 14 for immunohistochemical staining with MMP-9 antibody (n = 5 mice per group). Representative images of MMP-9 stained skin sections (100× magnification) from day 1 (A, B), day 7 (C, D) and day 14 (E, F) wounds from WT (A, C, E) and *Ripk3* ^-/-^ (B, D, F) mice are shown. MMP-9 is stained brown; ep, new epithelium; wb, wound bed. Scale bar = 100 μm. (G-H) RNA was extracted and cDNA was prepared from wound skin samples collected at the indicated days. MMP-9 (G) and Timp-1 (H) gene expression was analyzed by qPCR. Data expressed as mean ± SEM (n = 5 mice per group) and compared by one-way ANOVA using SNK test. *P < 0.05 versus WT at the indicated day.

Matrix metalloproteinases (MMP) are required for degradation of extracellular matrix during wound healing. MMP-9 is member of MMP family which is involved in inflammation, matrix remodeling, and epithelialization during cutaneous wound healing. As previous histological analysis of *Ripk3* ^-/-^ wounds showed defects in epithelialization, granulation tissue formation, collagen deposition, we immunostained the wounded skin tissues to examine MMP-9 expression. MMP-9 protein was expressed as early as day 1 post—wound in WT, while it was barely detected in *Ripk3* ^-/-^ wounds (Fig 5A and 5B). At 7 day post-wound, MMP-9 expression was increased compared to day 1 in both wounds; however, its expression was still lower in *Ripk3* ^-/-^ wounds compared to wounds in WT (Fig 5C and 5D). Even at day 14, when MMP-9 levels were down-regulated in both wounds from day 7, *Ripk3* ^-/-^ showed less MMP-9 in comparison to WT (Fig 5E and 5F). Overall, the intensity of MMP-9 expression in *Ripk3* ^-/-^ wounds is weaker than that in WT wounds during the entire healing process.

We further determined the mRNA expression levels of MMP-9 and its inhibitor, tissue inhibitor of metalloproteinases 1 (Timp-1), during the course of wound healing. Like MMP-9 protein expression pattern, its mRNA levels in WT wounds are increased as early as day 1, peaked at day 7 and dropped down by day 14 (Fig 5G). Although the levels of MMP-9 protein expression in *Ripk3* ^-/-^ wounds were lower than those in WT wounds, the levels and pattern of MMP-9 mRNA expression in *Ripk3* ^-/-^ wounds were comparable to those in WT wounds (Fig 5G). In contrast, the levels of Timp-1 mRNA in *Ripk3* ^-/-^ wounds were significantly higher than those in WT wounds through the 14-day healing course with an increase as early as day 1 (Fig 5H).

### RIPK3 deficiency delays angiogenesis and alters angiogenic growth factor expression

**Fig 6:**
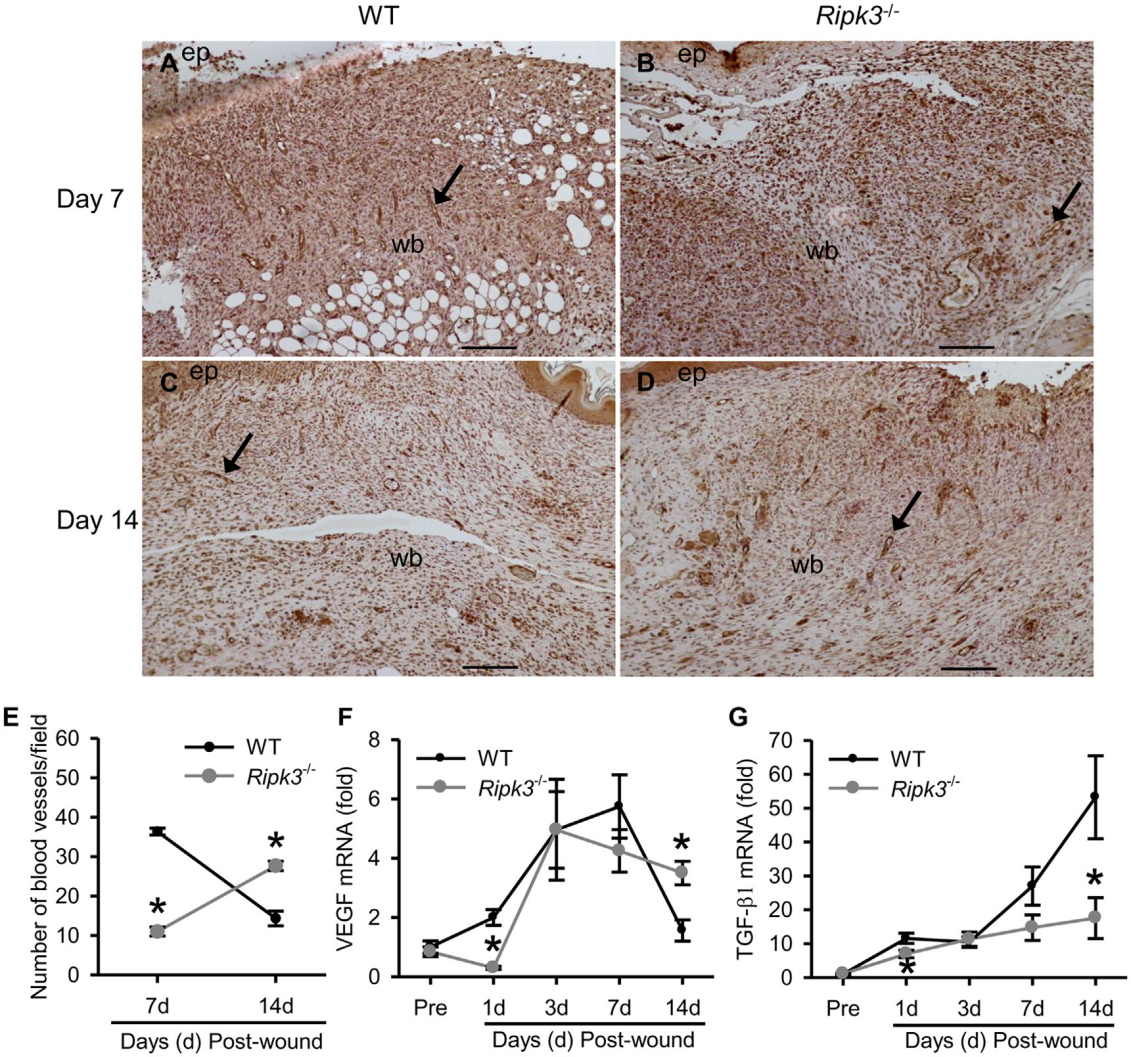
*Ripk3* ^-/-^ mice show delayed angiogenesis and altered pattern of growth factor induction. Skin samples from the wound sites of WT and *Ripk3* ^-/-^ mice were collected at the indicated days. (A-D) Skin samples were sectioned and immunostained with antibody against the endothelial cell junction molecule CD31. Representative images of CD31 stained skin sections (100× magnification) from day 7 (A, B), and day 14 (C, D) wounds from WT (A, C) and *Ripk3* ^-/-^ (B, D) mice are shown. CD31 is stained brown. Blood vessels were identified by CD31 positive endothelial cells lining the lumen and are depicted by arrows; ep, new epithelium; wb, wound bed. Scale bar = 100 μm. (E) Statistical evaluation of the density of CD31-positive blood vessels (expressed as number of vessels/field) in wound sections at day 7 and 14 post-wound. (F-G) Total RNA extracted from skin tissues were analyzed for mRNA expression of VEGF (F) and TGF-β1 (G) by qPCR. Data expressed as mean ± SEM (n = 3 (E) and 5 (F-G) mice per group for each time point) and compared by one-way ANOVA using SNK test. *P < 0.05 versus WT at the indicated day.

As histological analysis of *Ripk3* ^-/-^ wounds indicated delayed vascularization, so we performed immunostaining of endothelial cell junction molecule CD31 for the confirmation. At day 7, WT wounds had 3.3-fold more blood vessels stained with CD31, compared to the *Ripk3* ^-/-^ wounds (Fig 6A, 6B and 6E). However, at day 14, WT wounds only had a few areas with CD31 positive blood vessels (Fig 6C), indicating that they were in the maturation phase. By contrast, 14 day *Ripk3* ^-/-^ wounds still exhibited a lot of CD31-positive blood vessels (Fig 6D), 1.9-fold more compared to WT wounds (Fig 6E), indicative of delayed angiogenesis in *Ripk3* ^-/-^ wounds.

We also determined the gene expression of two key proangiogenic factors, VEGF and TGF-β1, in skin tissues by qPCR. As shown in Fig 6F, at day 1 VEGF expression was increased by 2-fold in WT wound tissue compared to pre-wound levels. In contrast, at day 1 VEGF levels in *Ripk3* ^-/-^ wound tissue were decreased to one third of the pre-wound levels which comes to a significant decrease by 85% compared to WT wounds (Fig 6F). At days 3 and 7 VEGF expression was upregulated comparably in WT and *Ripk3* ^-/-^ wounds. However, at day 14 VEGF levels dropped down close to day 1 levels in WT wounds whereas in the *Ripk3* ^-/-^ wounds VEGF levels were still 2.2-fold higher compared to WT wounds (Fig 6F). TGF-β1 expression in WT wounds was increased by 10-fold at day 1 to 50-fold at day 14 (Fig 6G). However, TGF- β1 expression remained low in *Ripk3* ^-/-^ wounds compared to WT wounds (Fig 6G), especially significant at day 1 (down by 40%) and day 14 (down by 67%). These results indicate that RIPK3 deficiency alters the temporal expression pattern of VEGF and TGF-β1 resulting into delayed angiogenesis and defected matrix formation after the wound.

### RIPK3 deficiency reduces fibroblast chemotaxis

**Fig 7:**
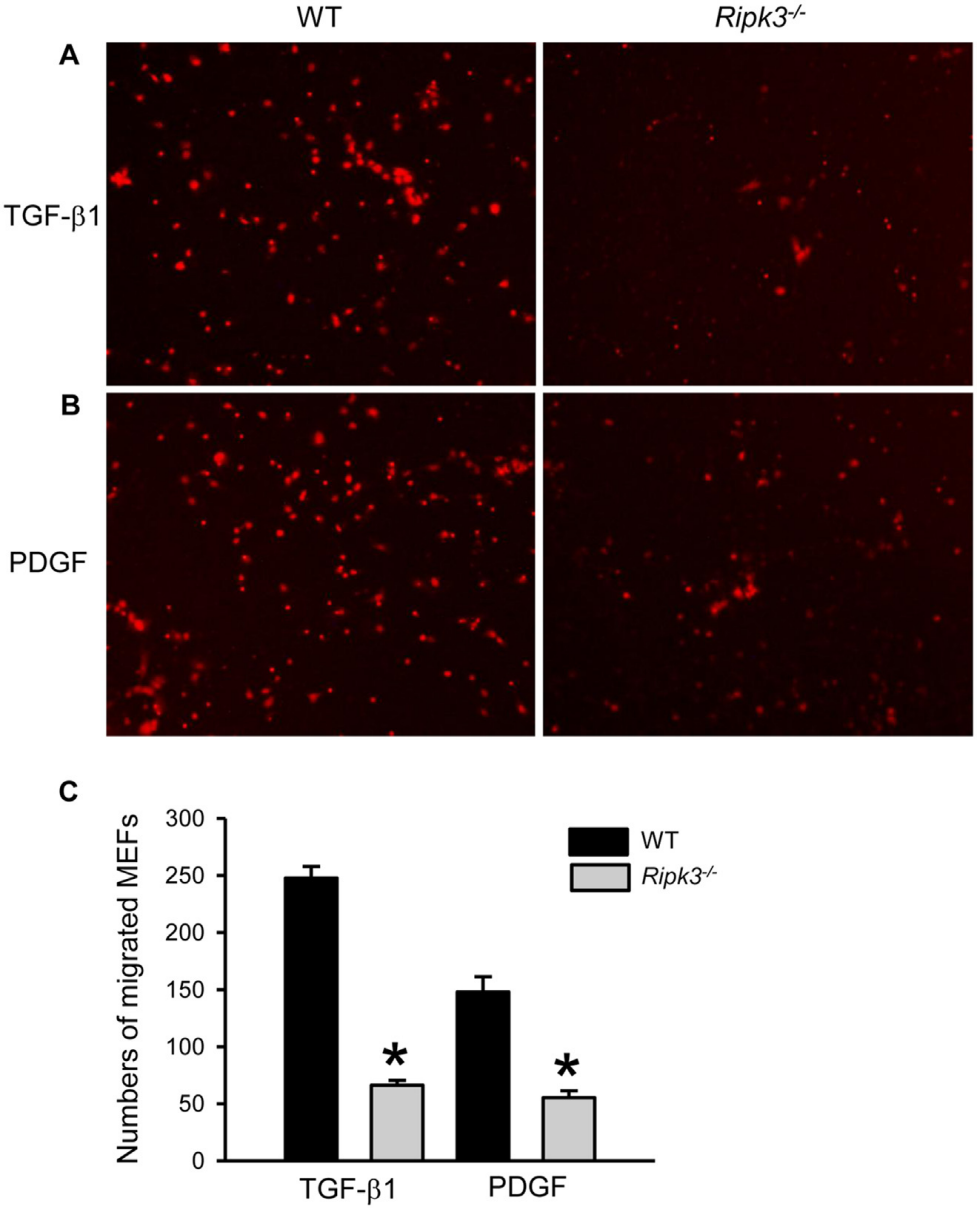
*Ripk3* ^-/-^ mice show reduced chemotaxis of fibroblasts in response to growth factors. Mouse embryonic fibroblasts (MEFs) were prepared from WT and *Ripk3* ^-/-^ mice and subjected to transwell migration assay as described in Materials and Methods with chemoattractants PDGF (A), and TGF-β1 (B). (C) The numbers of MEFs from WT and *Ripk3* ^-/-^ mice migrated toward the indicated chemoattractant. The experiment was repeated three times and data expressed as mean ± SEM (n = 3–4 per group per experiment) and compared by Student’s *t*-test. *P < 0.05 versus WT at the indicated day.

Simultaneously with angiogenesis, fibroblast migration and collagen deposition forms granulation tissue. As the inflammatory phase of wound healing nears completion, macrophages and platelets release fibroblast growth and chemotactic factors to activate fibroblasts which then migrate into the wound site. As histological analysis of *Ripk3* ^-/-^ wounds showed irregularities in granulation tissue formation and collagen deposition, we performed a chemotaxis assay with WT and *Ripk3* ^-/-^ MEFs to compare their migration capability. The numbers of MEFs from *Ripk3* ^-/-^ mice migrated towards TGF-β1 and PDGF were significantly reduced by 73% and 63%, respectively, compared to MEFs from WT mice (Fig 7).

## Discussion

Wound healing is a complex and highly regulated process comprising of overlapping events such as inflammation, proliferation and tissue remodeling. Inflammatory phase is characterized by cell proliferation and migration; proliferative phase is marked by collagen deposition and angiogenesis; and maturation phase involves resolution of inflammation and scar maturation [1]. Impaired or delayed healing of cutaneous wounds as a result of disruption at any step in the wound healing process can lead to the development of chronic non-healing ulcers [6, 7]. A better understanding of molecules involved during wound healing process is needed to discover novel molecular targets which may provide new therapeutic strategies for wound healing. RIPK family members play important roles in inflammation and cell-death [12]. RIPK4 is an established controller of cutaneous wound repair [13–15] whereas RIPK3 is known to be highly expressed during wound healing [13]. While inhibition of RIPK3 has been shown to be beneficial in many tissue injury models [22, 29–31], Chan et al. hinted that it may result in the potential impairment of the wound healing process [32]. However, RIPK3-deficient mice are developmentally normal [27] and role of RIPK3 in wound healing has not been investigated.

In the present study, using a mouse model of cutaneous wound healing, we demonstrated for the first time that RIPK3 deficiency significantly delayed the wound closure rate over the 14-day course. RIPK3 deficiency also impaired the quality of healing wounds characterized by compromised reepithelialization, delayed angiogenesis, irregular granulation tissue and collagen deposition in comparison with WT wounds. RIPK3 also played a crucial role in altering neutrophil trafficking and inflammatory cytokine pattern during early wound healing. *Ripk3* ^-/-^ wounds showed an initial delay in neutrophil infiltration and chemokine production with a subsequent increase later at day 3 compared to WT wounds during the early inflammatory phase. Impaired wound healing in *Ripk3* ^-/-^ mice was also associated with decreased MMP-9 protein expression, increased Timp-1 mRNA expression, delayed CD31 staining and VEGF production as well as reduced TGF-β1 levels. Furthermore, *Ripk3* ^-/-^ MEF showed significantly lower chemotactic activity toward growth factors TGF-β1 and PDGF than WT MEF.

The wound closure rate in *Ripk3* ^-/-^ mice was significantly delayed by day 7; however, by day 14 both *Ripk3* ^-/-^ as well as WT wounds reached almost complete closure. Wound healing in WT mice progressed normally with mature granulation tissue formation via proliferating inflammatory cells and fibroblasts with subsequent collagen deposition. The major defects seen in the histological analyses of the *Ripk3* ^-/-^ wounds at day 7 were delayed reepithelialization, vascularization and collagen deposition, suggesting that RIPK3 is critical for these processes. Even at day 14 when the wound closure of *Ripk3* ^-/-^ wounds was comparable to WT, histological analyses showed delayed vascularization and irregular granulation tissue and collagen deposition pattern in the *Ripk3* ^-/-^ wounds. This can be explained by the wound contraction phenomenon seen in loose-skinned rodents. The wound closure quantified as a percent change in wound surface area compared to the original wound’s size is a measure of contraction which is mediated by myofibroblasts. The full thickness wounds such as those created in our study heal by a combination of contraction, reepithelialization, and dermal reconstitution [33]. So even if contraction is comparable between WT and *Ripk3* ^-/-^ wounds at day 14, delayed angiogenesis and defects in the organization of granulation tissue and collagen would still serve as a measure for the delay in healing.

The inflammatory cell infiltration into the wounded tissue was comparable in WT and *Ripk3* ^-/-^ wounds at days 7 and 14 post-wound (data not shown). However, inflammatory response in the early stages of wound healing is needed for the recruitment of neutrophils that clear potential bacterial contamination and produce pro-inflammatory cytokines to activate local fibroblasts and keratinocytes [1]. This inflammation resolves on its own but if it goes uncontrolled, it leads to impaired healing [6, 8]. We also found neutrophil infiltration as early as day 1 which decreased at day 3 in WT cutaneous wounds. On the other hand *Ripk3* ^-/-^ wounds showed lesser neutrophil infiltration at day 1 and more neutrophil infiltration at day 3 than WT wounds. In a previous study, RIPK3 expression is strongly induced at day 1 to 3 during wound healing, which is prominently detected in dermal immune cells and then returns to barely detectable levels [13]. The high expression of RIPK3 in the early inflammatory phase (1–3 day after injury) of wound healing further supports the important role of RIPK3 in regulating the timing of neutrophil infiltration in wound healing.

IL-6, TNF-α and IL-1β have been reported to be mainly secreted by neutrophils and rapidly induced post-wound during murine wound healing [34]. KC, a murine homologue of human IL-8, is a major neutrophil chemotactic factor and a critical inflammatory mediator. Consistent with this, we also observed a rapid and early induction of IL-6, KC, IL-1β and TNF-α in the wounds of WT mice. However, at day 1 IL-6, KC, and IL-1β levels in *Ripk3* ^-/-^ wounds were much lower than WT wounds. In addition, at day 3 *Ripk3* ^-/-^ wounds showed elevated IL-6, KC, and TNF-α levels than WT wounds which would again suggest defective wound healing. Thus, the expression patterns of IL-6, KC, TNF-α and IL-1β were in consistence with the neutrophil infiltration pattern, showing the difference between WT and *Ripk3* ^-/-^ wounds at the early phase in days 1 and 3, but no significant difference at the later phases in days 7 and 14.

Wound healing requires several processes similar to development such as cell migration, extra-cellular matrix degradation, and tissue reorganization. Keratinocytes at the edge of the wound have to migrate to re-epithelialize the wound surface and then the fibrin-rich provisional matrix that is laid down following wounding must be removed for which MMP are required. MMP-9 expression has been shown to increase in the earlier inflammatory phase and decrease during later phases of wound healing [35]. We also observed MMP-9 expression in the WT wounds as early as day 1 which further increased by day 7 and dropped down by day 14. MMP-9 protein levels in *Ripk3* ^-/-^ wounds followed the similar pattern as WT but were lower compared to WT wounds at all time-points. However, the mRNA levels of MMP-9 in WT and *Ripk3* ^-/-^ wounds did not correspond to their protein levels. Such discrepancy between protein and mRNA levels in MMP-9 has also been reported in another study [36]. The lower MMP-9 levels in *Ripk3* ^-/-^ wounds were also supported by an increase in the expression of the MMP-9 inhibitor Timp-1 observed in *Ripk3* ^-/-^ wounds. We also measured the mRNA levels of MMP-2 by qPCR. The expression levels and pattern of MMP-2 in both *Ripk3* ^-/-^ and WT wounds were comparable, showing an increase as early as day 1, peaked at day 7 and remained high by day 14 (data not shown). Although keratinocyte proliferation plays a role in the healing process, RIPK3 may not have a direct effect on regulating this character since its expression was absent or very weak in epidermal keratinocytes [13]. In addition, MMP-9-deficient mice have been reported to display delayed wound healing and defects in keratinocyte migration and collagen fibrillogenesis leading to delayed reepithelialization and irregular matrix remodeling in the later stages of wound healing [37]. Accordingly, our data showed that the *Ripk3* ^-/-^ mice also displayed delayed reepithelialization and irregular matrix remodeling which could be due to decreased MMP-9 expression.

Angiogenesis is a critical factor in successful wound repair and is known to be tightly regulated in a complex interplay of angiogenic and angiostatic growth factors. One of the main angiogenic growth factors, VEGF, has a pleiotropic role in tissue repair via neovascularization, reepithelialization, and regulation of extracellular matrix [38]. VEGF promotes the early stages of angiogenesis i.e., vascular dilation, permeability, migration, and proliferation [38, 39]. In consistence with a previous report, we also observed maximal VEGF mRNA expression between 3 and 7 days post-wound which is the period of granulation tissue formation and a decline in VEGF mRNA to basal levels after 14 days in WT mice [40]. VEGF levels in *Ripk3* ^-/-^ wounds were significantly lower at day 1 and significantly higher at day 14 than WT wounds, which would indicate a delay in early angiogenesis as well as defected matrix formation. TGF-β1 is multifunctional and plays role in all three phases of wound healing. TGF-β1 encourages the inflammatory cell infiltration, angiogenesis, fibroblast proliferation, migration, and extracellular matrix production [41, 42]. In our study, we also observed the induction of TGF-β1 after wounding at all time points during the 14 day course of wound healing in the WT mice. The *Ripk3* ^-/-^ mice showed reduced TGF-β1 levels which were very significant at days 1 and 14, which would again indicate towards delayed angiogenesis as well as defects in early inflammatory phase and late tissue remodeling phase.

Angiogenesis during wound healing is accompanied by fibroblast migration into the wound and subsequent collagen deposition. Growth factors, especially TGF-β1 and PDGF presumably stimulate fibroblasts of the tissue around the wound to proliferate, express appropriate integrin receptors, and migrate into the wound space [1]. Fibroblasts numbers at the wound site peak 7–14 days post-wound [43]. The fibroblasts are responsible for the synthesis, deposition, and remodeling of the extracellular matrix [1]. Our chemotaxis assay with WT and *Ripk3* ^-/-^ MEFs showed lower chemotactic activity of *Ripk3* ^-/-^ fibroblasts toward growth factors TGF-β1 and PDGF compared to WT fibroblasts. This reduced fibroblast migration, in part, could be a reason for irregularities in granulation tissue formation and collagen deposition seen in *Ripk3* ^-/-^ wounds.

In summary, we demonstrated defective wound healing in RIPK3-deficient mice. Our study suggests that RIPK3 is required to control the temporal order at multiple steps, including neutrophil trafficking, inflammatory cytokine production, reepithelialization, angiogenesis, fibroblast migration, granulation tissue formation and collagen deposition, for normal progression and good quality of wound healing.

## Supporting Information

S1 ARRIVE Checklist The ARRIVE Guidelines Checklist. (PDF)
